# Reproducibility of Skin Temperature Response after Cold Stress Test Using the Game Ready System: Preliminary Study

**DOI:** 10.3390/ijerph18168295

**Published:** 2021-08-05

**Authors:** Jose Ignacio Priego-Quesada, Alexis Gandia-Soriano, Maria Teresa Pellicer-Chenoll, Ignacio Catalá-Vilaplana, Jose Luis Bermejo-Ruiz, Alberto Encarnación-Martínez, Rosario Salvador-Palmer, Rosa Cibrián Ortiz de Anda

**Affiliations:** 1 Research Group in Sports Biomechanics (GIBD), Department of Physical Education and Sports, Universitat de València, 46010 Valencia, Spain; ignacio.catala@uv.es (I.C.-V.); Alberto.encarnacion@uv.es (A.E.-M.); 2 Research Group in Medical Physics (GIFIME), Department of Physiology, Universitat de València, 46010 Valencia, Spain; alexisgandiasoriano@gmail.com (A.G.-S.); Rosario.Salvador@uv.es (R.S.-P.); rosa.m.cibrian@uv.es (R.C.O.d.A.); 3 Department of Physical Education and Sports, Universitat de València, 46010 Valencia, Spain; M.Teresa.Pellicer@uv.es (M.T.P.-C.); J.Luis.Bermejo@uv.es (J.L.B.-R.)

**Keywords:** infrared thermography, dynamic thermography, cooling, medicine, posterior leg, cryotherapy

## Abstract

The objective of this preliminary study was to determine the reproducibility of lower limbs skin temperature after cold stress test using the Game Ready system. Skin temperature of fourteen participants was measured before and after cold stress test using the Game Ready system and it was repeated the protocol in four times: at 9:00, at 11:00, at 19:00, and at 9:00 h of the posterior day. To assess skin temperature recovery after cold stress test, a logarithmic equation for each region was calculated, and constant (β_0_) and slope (β_1_) coefficients were obtained. Intraclass correlation coefficient (ICC), standard error (SE), and within-subject coefficient of variation (CV) were determined. No differences were observed between measurement times in any of the regions for the logarithmic coefficients (*p* > 0.38). Anterior thigh (β_0_ ICC 0.33–0.47; β_1_ ICC 0.31–0.43) and posterior knee (β_0_ ICC 0.42–0.58; β_1_ ICC 0.28–0.57) were the regions with the lower ICCs, and the other regions presented values with a fair and good reproducibility (ICC > 0.41). Posterior leg was the region with the better reproducibility (β_0_ ICC 0.68–0.78; β_1_ ICC 0.59–0.74; SE 3–4%; within-subject CV 7–12%). In conclusion, cold stress test using Game Ready system showed a fair and good reproducibility, especially when the posterior leg was the region assessed.

## 1. Introduction

The assessment of skin temperature has been used in medicine for diagnosis or evaluation of different pathologies such as those that have an altered peripheral circulation as diabetes or Raynaud’s phenomenon, for inflammatory diseases as arthritis, for traumatic injuries, or for malignant diseases such as breast cancer, among others [1,2,3]. These applications are based on the idea that skin temperature is affected by the inflammation in underlying tissues, by the higher metabolic activity resulted from angiogenesis, by nerve dysfunctions, or by the alteration of skin blood flow [1,3,4]. However, the assessment applications of skin temperature have not been limited to medicine and have also been used in other contexts such as the study of thermoregulation during exercise, the evaluation of the effect of clothing, or the assimilation of the athlete’s training load, among others [4,5]. However, for some of these applications, baseline skin temperatures could not provide enough information, and Cold Stress Tests (CST) could improve the sensitivity of the prediction and diagnosis [2,3].

CST have been applied for hypertension and diabetic population [6,7] to assess the severity of cold sensitivity [8,9], assess physiological stress the day after a marathon [10], or diagnose Raynaud pathology [11]. CST consist of cooling the skin to evaluate the posterior vascularization capacity [12,13]. During CST, a higher sympathetic activity results in peripheral vasoconstriction, which facilitates skin temperature reduction [14,15,16]. After finishing the CST, skin vasodilation increases resulting in a rewarming of skin temperature [14,15,16]. Although skin temperature rewarming depends mainly on skin blood flow, it can also occur passively from the environment and deeper tissues [13] and is influenced by the duration of the cooling phase [17].

Although there is a high intersubject variability response to CST [18], good reproducibility of skin temperature after CST has been observed by different studies due to a lower within-subject variability [8,15,19]. However, this reproducibility is not generalizable to all the body regions [20]. For example, O’Brien [15] observed reproducible finger skin temperatures in the nail bed, but not in the pad. These studies assessed hands and foot, being the most used method the immersion of these extremities in cold water [8,15,19]. Therefore, it is necessary to determine the reproducibility for specific regions with new methodologies of CST. In this sense, recently, a system of controlled pressure with cryotherapy (Game Ready^®^, Avanos Medical Inc. Company, Alpharetta, GA, USA) has been used to assess skin temperature of lower limbs after CST [10].

Game Ready^®^ is a system that combines a control unit filled with ice and water that pumps the water constantly to the circumferential wraps (by ducts inserted into the tissue) to apply cold and intermittent pneumatic compression [21]. It has been clinically applied to mitigate pain and inflammation after an injury or during the recovery from different surgeries such as total knee arthroplasty [22], lumbar spinal surgery [23], or hip arthroscopy [24], among others. Although its application for CST is scarce, we decided to investigate its use for CST due to its combination with pressure, allowing the cooling to be more homogeneous over the entire surface. In addition, another advantage would be that the risk of the skin being wet is avoided, as can happen with other CST methods, a critical issue in thermographic analysis [25].

The objective of this preliminary study was to determine the reproducibility of lower limbs skin temperature after CST using the Game Ready system. Considering that this system could homogenize better the conditions than the water immersion methodology, and the regions assessed are less peripherical than hands and foot, which could improve its reproducibility [26], it was hypothesized that skin temperature rewarming after CST would present a good reproducibility.

## 2. Materials and Methods

### 2.1. Participants

Fourteen (4 females and 10 males) physically active participants volunteered to participate in the study (mean ± standard deviation): age 36 ± 5 years, height 174 ± 7 cm, body mass 77.0 ± 11.6 kg, body fat percentage 21 ± 8%, muscle mass percentage 76 ± 10%. Inclusion criteria were to be physically active with a reported training schedule of at least 3 physical activity sessions/week. Exclusion criteria were a history of lower limb injuries within the last month, suffering from heart failure, neurological or musculoskeletal disorders affecting normal locomotion, and taking medication that interferes with stability during running. All participants signed a written consent form, and the University Ethics Committee approved the study. The instructions provided to the participants to control the factors that can affect skin temperature were to avoid exercise, large meals, ointments, and cosmetics in the days of the measurements; to avoid smoking, drinking alcohol, caffeine, or other stimulant beverages from 12 h before to finishing the measurements; and to avoid physiotherapy treatments, sunbathing and high-intensity physical activity from 24 h before to finishing the measures. The participants confirmed their compliance with all these instructions.

### 2.2. Design

To understand the effect of the system or the intra-subject physiological variability, measures were performed in different moments (morning vs. afternoon vs. different day). Therefore, participants were assessed in four times: (1) at 9:00, (2) at 11:00 (to have a measurement in the same day as time 1 and also at morning), (3) at 19:00 (to have a measurement in the same day as time 1 but at afternoon), and (4) at 9:00 of the posterior day (to have a measurement in the same hour as time 1 but in a different day). In the first measurement, body composition variables were obtained using a bioelectrical impedance system (Tanita BC-545N, Tanita Corp., Tokyo, Japan). In all the measurements, participants stood in an upright resting position (men wearing underpants and women in panties) for 10 min to adapt to the temperature of the room [27]. Once the thermal room adaptation was completed, a thermography image of the preferred lower limb was obtained, CST was performed, and then thermographies were taken again for three minutes (30, 60, 120, and 180 s) after finishing CST of the preferred lower limb. These times were taken after observing in a pilot that after removing the wrap and the participant positioned for the image, 30 s was the minimum time that could be assured to take the first image. Because the skin temperature recovery describes a logarithmic curve, after the minute, the thermal recovery rate is slower, and thermal images can be taken to the minute. CST had a duration of 3 min using an electronic cryotherapy system (Game Ready GRPro 2.1, Avanos Medical Inc. Company, Alpharetta, GA, USA) while the participants were lying supine [10]. The circumferential wrap used was the “full leg boot wrap” model for colling the entire lower limb. The Game Ready system was configured to the lowest temperature (between 0 and 3 °C) with moderate pressure for the wrap (intermittent pneumatic compression between 5 and 50 mm Hg considering manufacturer information) [10].

Room temperature was controlled with an air conditioning system and environmental conditions were recorded using a thermo-hygrometer (Digital thermo-hygrometer, TFA Dostmann, Germany) with the following values: 19.7 ± 1.8 °C room temperature and 38 ± 3% of relative humidity.

### 2.3. Skin Temperature

Skin temperature was assessed using an infrared thermography camera (E-60bx, Flir Systems Inc., Wilsonville, OR, USA) with a sensor array size of 320 × 240, noise equivalent temperature difference (NETD) < 0.05 °C, instantaneous field of view of 1.36 mrad, and measurement uncertainty of ±2 °C. Camera calibration was checked one week before the measurements using a blackbody (BX-500 IR Infrared Calibrator, CEM, Shenzen, China) with a target emissivity of 0.95, resolution of 0.1 °C, and measurement uncertainty of ±0.8 °C. A Thermographic Imaging in Sports and Exercise Medicine checklist was used to confirm that all the essential aspects of the thermographic protocol were attended [28]. The camera was turned on 10 min before the measurements and it was positioned 3 m from the participant. An anti-reflective panel was placed behind the participant to minimize the influence of the radiation reflected [1]. The reflected temperature was measured according to the standard method ISO 18434-1:2008. Measurements were taken in a room absent of sunlight and 5 m away from any electronic equipment (except the Game Ready system).

**Figure 1:**
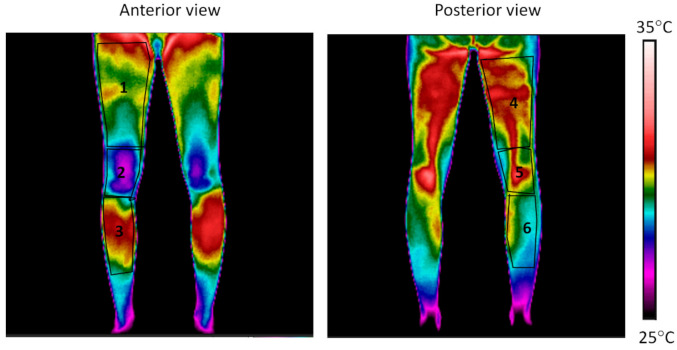
Regions of interest assessed in the preferred lower limb: (1) anterior thigh, (2) knee, (3) anterior leg, (4) posterior thigh, (5) posterior knee, and (6) posterior leg.

**Figure 2:**
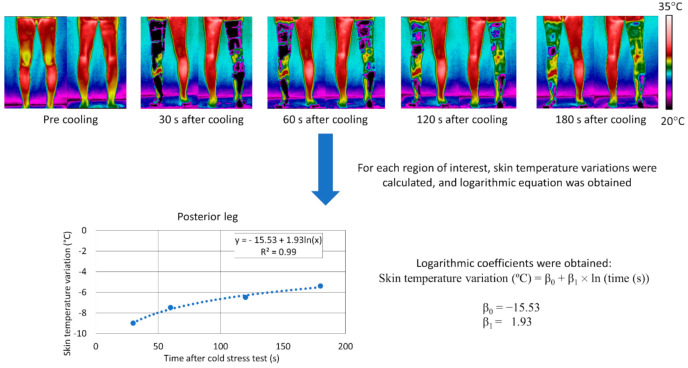
Schematic representation of the process to obtain the logarithmic coefficients to assess cold stress test.

The mean temperature of 6 Regions of Interest (ROIs) of the preferred lower limb (Figure 1) with an emissivity of 0.98 [28,29] was obtained using thermography software (Thermacam Researcher Pro 2.10 software, FLIR, Wilsonville, OR, USA). The same researcher defined ROIs to ensure consistent analysis across the sessions considering body segments. The temperature obtained after the CST (30, 60, 120, and 180 s after) was subtracted from the one obtained before the cold stress test to assess the skin temperature variation. To assess skin temperature recovery after CST, data were placed in an Excel spreadsheet and a logarithmic equation for each participant, measurement time, and ROI was calculated, and constant (β_0_) and slope (β_1_) coefficients were obtained (Figure 2) [10]:

Skin temperature variation (°C) = β_0_ + β_1_ × ln (T)

where the β_0_ and β_1_ are the constant and slope coefficients of the equation, respectively; ln is the natural logarithm; T is the time lasted after the cold stress in seconds; and skin temperature variation is the difference between the skin temperature at T respect with pre-cooling moment.

### 2.4. Statistical Analysis

Statistical analysis was performed using RStudio (version 1.2.5033). The normality of the logarithmic coefficients was confirmed using the Shapiro–Wilk test (*p* > 0.05). Then, for each ROI, repeated-measures analysis of variance with Bonferroni post hoc test were applied with a within-subject factor (measurement time: 9:00 vs. 11:00 vs. 19:00 vs. 9:00 of the posterior day). Significance level was set at *p* < 0.05. The intraclass correlation coefficient (ICC), based on a single rater-measurement, absolute-agreement, and 2-way random-effects model, was calculated at each ROI, between different measurement times comparisons: 1-between measurements in the same morning (9:00–11:00), 2-between the measurement at morning and at afternoon (9:00–19:00), 3-between the measurement at morning and 24 h post (9:00–9:00 day 2), and 4-between all measurement times. ICC values were classified as excellent reproducibility (1.00 to 0.75), good reproducibility (0.74 to 0.60), fair reproducibility (0.59 to 0.40), and poor reproducibility (0.39 to 0.00) [30]. Standard error (SE) was provided as a measure of uncertainty. Finally, within-subject and between-subject coefficient of variation (CV) was calculated [31].

## 3. Results

**Figure 3:**
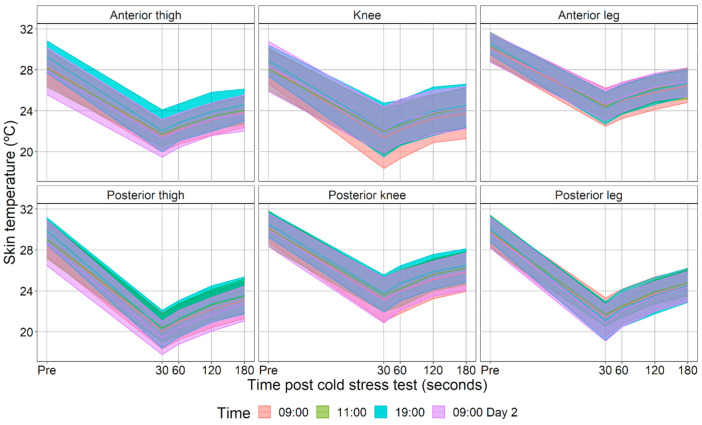
Mean and standard deviation of the evolution of skin temperature before (Pre) and 30, 60, 120, and 180 s after cold stress test at each measurement time.

**Figure 4:**
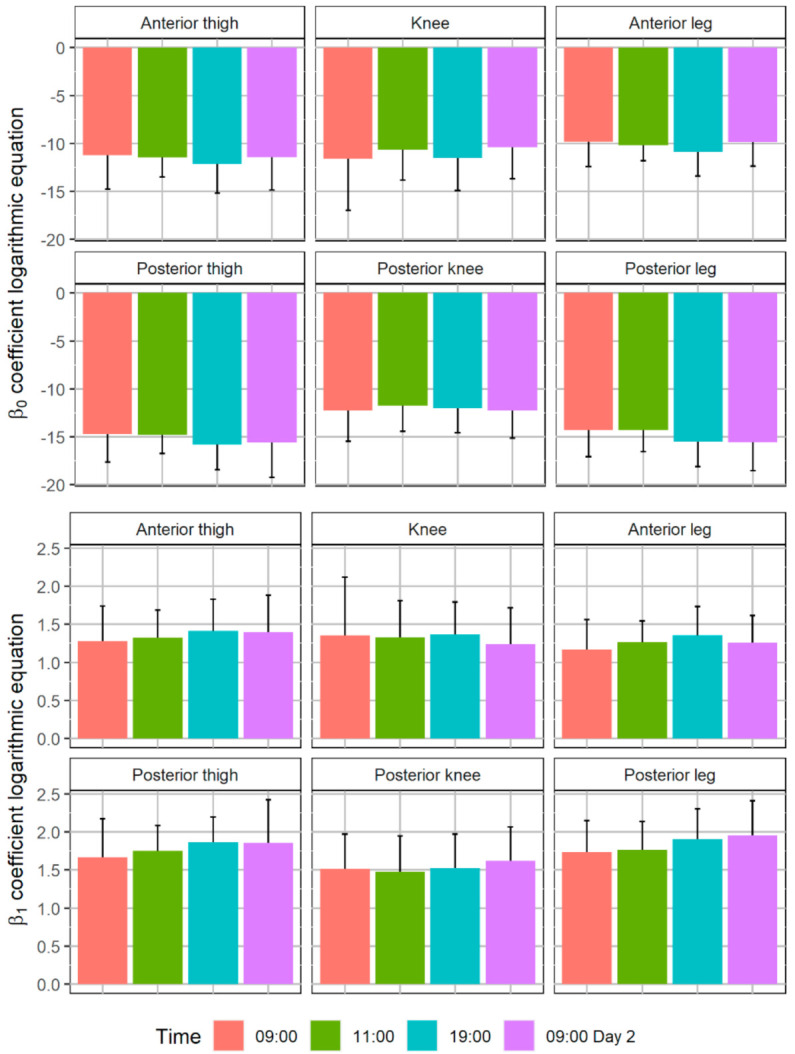
Mean and standard deviation of the coefficients of the logarithmic equations obtained with the cold stress test: Skin temperature variation (°C) = β_0_ + β_1_ × ln (T), where β_0_ and β_1_ are the constant and slope coefficients of the equation, respectively; ln is the natural logarithm; T is the time lasted after the cold stress in seconds; and skin temperature variation is the difference between the skin temperature at T respect with pre-cooling moment. No differences were observed between measurement times in each region of interest (*p* > 0.05).

Figure 3 shows the skin temperature data used for the calculation of the logarithmic equations. The R^2^ of the logarithmic equations obtained for each participant demonstrated their good adjustment: anterior thigh 0.95 ± 0.05, knee 0.94 ± 0.06, anterior leg 0.97 ± 0.03, posterior thigh 0.97 ± 0.04, posterior knee 0.96 ± 0.05, and posterior leg 0.98 ± 0.02. Figure 4 shows the logarithmic equations values obtained. No differences were observed between measurement times in any of the ROIs for the β_0_ (anterior thigh *p* = 0.89, knee *p* = 0.80, anterior leg *p* = 0.61, posterior thigh *p* = 0.68, posterior knee *p* = 0.96, and posterior leg *p* = 0.39) and β_1_ (anterior thigh *p* = 0.81, knee *p* = 0.93, anterior leg *p* = 0.59, posterior thigh *p* = 0.61, posterior knee *p* = 0.87, and posterior leg *p* = 0.43).

Table 1 shows the ICC, SE, and CV values obtained. Generally, comparing the reproducibility obtained between the different ROIs, the ROIs with the lower ICC were the anterior thigh with values between poor and fair reproducibility and (β_0_ ICC 0.33–0.47; β_1_ ICC 0.31–0.43) and the posterior knee with fair reproducibility (β_0_ ICC 0.42–0.58; β_1_ ICC 0.28–0.57). Posterior thigh presented between fair and good reproducibility (β_0_ ICC 0.53–0.69; β_1_ ICC 0.38–0.64), and knee and anterior leg between fair and excellent reproducibility (β_0_ ICC 0.49–0.73; β_1_ ICC 0.42–0.77). Posterior leg was the region with the better reproducibility (β_0_ ICC 0.68–0.78; β_1_ ICC 0.59–0.74). Comparing the different measurement times at each ROI, knee, anterior leg, and posterior thigh presented the highest reproducibility in the comparison between 9:00 and the measurement performed after 24 h (β_0_ ICC 0.69–0.73; β_1_ ICC 0.64–0.77). The SE of β_0,_ presented values between 0.3 and 0.8 with a mean value of 0.5, and the SE of β_1_ between 0.05 and 0.12 with a mean value of 0.08. The percentage of the SE respect the mean value of the β_0_ coefficient of each ROI was anterior thigh 5%, knee 7%, anterior leg 4%, posterior thigh 3%, posterior knee 4%, posterior leg 3%, and for all the ROIs 4%. The percentage of the SE respect the mean value of the β_1_ coefficient of each ROI was: anterior thigh 6%, knee 8%, anterior leg 5%, posterior thigh 5%, posterior knee 5%, posterior leg 4%, and for all the ROIs 5%. Generally, β_1_ presented lower reproducibility than β_0,_ showing also higher SE (4% vs. 5%) and within-subject CV (β_0_ CV 7–20%; β_1_ CV 9–27%). The between-subject CV was higher than the within-subject CV (16–47% vs. 7–27%). Finally, the ROIs that presented the lowest within-subject CV was posterior leg (7–12%), and the ROIs with the highest values were the knee (17–27%) and the anterior thigh (15–25%).

## 4. Discussion

The objective of this preliminary study was to determine the reproducibility of lower limbs skin temperature after CST using the Game Ready system. Therefore, participants were measured in four different measurement times: 9:00, 11:00, 19:00, and 9:00 of the posterior day. Coefficients of the logarithmic equation that describe skin temperature recovery after CST were obtained. The main results were that (1) no differences were observed between measurement times in any of the ROIs for the coefficients of logarithmic equation; (2) anterior thigh and posterior knee were the ROIs with the lower ICC with a poor and fair reproducibility, and the rest of the ROIs generally presented values with a fair and good reproducibility; and (3) posterior leg was the region with the better reproducibility and lower uncertainty.

Although a reproducibility between fair and good was obtained for most of the ROIs, anterior thigh and posterior knee presented lower values than the others, which would be something important to consider for future studies. Zaproudina et al. [26] suggested that the reproducibility of skin temperature between different days could be affected by the technical characteristics of the infrared thermography camera, but also by the variability of participants’ skin blood flow. Regarding the anterior thigh, although evaluators tried to position the Game Ready system to cover up to the crotch, it might not fit perfectly for some participants, resulting in lower reproducibility. In this sense, in a previous study, the authors did not analyze anterior and posterior thigh ROIs because the Game Ready system did not cover in all cases with the same proportion of the region surface [10]. However, note that this lower reproducibility was not observed in the posterior thigh. Another explanation that could be added to the previous one is the contact of the wrap sheath with the lower limb. Although the Game Ready system was configured with medium compression to homogenize its contact with the skin, the posterior regions were also supported on the table by the supine position, which could increase the pressure and improve its contact. All these explanations are attributable to the ROI with the better reproducibility and lower uncertainty, the posterior leg, which was covered completely by the Game Ready system, and where the pressure could be positively affecting. To validate this hypothesis, a future study could analyze the reproducibility of the system using different compression levels and different postures (supine vs. prone). In the posterior knee case, the lower reproducibility of this region may be due to variability in blood flow, as the temperature of the popliteal region is mainly due to the volume of blood circulating through the popliteal artery that branches to the anterior tibial artery, the posterior tibial artery and the peroneal artery [32].

Within-subject CV is the statistical parameter used for most of previous studies and allow to compare reproducibility results. Considering the region of the posterior leg, which obtained the best results for all the parameters assessed (IC, SE, and CV), the within-subject CV of the logarithmic coefficients had values of 7–12%. These results are in agreement with previous studies that suggested good reproducibility of their CST assessed [8,15,33]. These studies presented within-subject CV, during hands and foot cold water immersion, between 8 and 10% for finger skin temperature [33], between 3 and 11% of absolute values of skin temperature post immersion [8], between 9 and 12% for nail bed region [15], and 17–21% for pad region [15].

A better reproducibility was observed in the comparison between 9:00 and the measurement performed after 24 h in some of the ROIs (knee, anterior leg, and posterior thigh). This result could be evidencing the effect on the reproducibility of the circadian cycle and increased daily activity with respect to the first measurement. The circadian rhythm was suggested as an important influence factor on skin temperature [34,35], due to its incremental effect on blood flow extremities during the day [36]. In this sense, higher skin temperatures (~1 °C) were observed at the afternoon than at the morning in thigh and legs [35]. Therefore, this should be an aspect to take into account in future research, carrying out the evaluations of the different study conditions at the same time of the day.

β_1_ presented lower reproducibility than β_0_, also showing higher SE and within-subject CV. β_0_ could be associate with the capacity of the Game Ready system to cold down skin temperature and the peripheral vasoconstriction state of the person assessed. If a person has a state of more significant peripheral vasoconstriction, it will facilitate this reduction in temperature [6,10,37]. On the other hand, β_1_ is related to the rewarming speed and, therefore, peripheral vasodilation capacity [6,38]. For this reason, it is logical that β_0_ has a higher reproducibility and lower uncertainty as it depends to a greater extent on the cooling system, while β_1_ depends mainly on the physiological variability of the participant.

The important advantage of CST has been evidenced by different pathologies and applications such as breast cancer [39], hyperthyroidism [40], hypertension [7], diabetes [6], cold sensitivity [9], Raynaud pathology [11], or physiological stress the day after a marathon [10]. Most of these applications are based on hands and water immersion, and the assessment of full lower limbs is less assessed due to its methodological difficulties. The use of systems of cryotherapy combined with intermittent pneumatic compression to the wrap such as the one used in the present study facilitate this type of analysis, as they are fast, logistically comfortable, and do not wet the skin, which would be a complication for temperature measurement using infrared thermography [25]. The results of the present study suggest the applicability of this type of CST, although if it is carried out with the same method used, the results in the posterior leg will be more sensitive, and less in the rest of the regions due to its lower reproducibility, higher uncertainty, and more within-subject variability. This is in agreement with a previous study that showed that the posterior leg was the only region in which the coefficients of logarithmic equation showed differences 24 h after running a marathon [10]. Finally, another practical application result was the higher between-subject variability compared with the within-subject variability. This suggests that, whenever possible, crossover experimental designs should be performed rather than independent group comparison.

This investigation was considered preliminary due to the sample size, only one type of system configuration was assessed in terms of temperature, pressure, and cold application time, and because the results suggest that the effect of other postures during cooling should be investigated. The main limitation of this preliminary study was that the lack of skin blood flow assessment that could help to interpretate if variability was due to CST methodology or due to the participant’s physiology. Moreover, although instructions were provided to the participants to avoid confounding factors, we cannot be sure that some participants did not follow any of the instructions and therefore affect reproducibility results. The Game Ready system can be used with different wrap models to cool specific regions, so future studies need to evaluate reproducibility in other body regions. Moreover, a future study would monitor wrap temperature continuously using contact sensors to assess the system’s reproducibility.

## 5. Conclusions

This preliminary investigation suggests that CST using the Game Ready system showed a good reproducibility, especially when the posterior leg was the region assessed. Therefore, its application can be recommended when it is necessary to evaluate the skin temperature of the lower limbs after CST. However, anterior thigh and posterior knee presented lower reproducibility, and these ROIs should be considered with caution when future studies will assess it.

## Figures and Tables

**Table 1:** Intraclass correlation coefficients (IC), standard error (SE), and within-subject and between-subject coefficient of variation (CV) of the measurement times comparisons, considering the coefficients of the logarithmic equations obtained with the cold stress test: Skin temperature variation (°C) = β_0_ + β_1_ × ln (time after the cold stress test (s)).

	ICC				SE				Within-Subject CV (%)				Between-Subject CV (%)			
	9:00–11:00	9:00–19:00	9:00–9:00 Day 2	All Moments	9:00–11:00	9:00–19:00	9:00–9:00 Day 2	All Moments	9:00–11:00	9:00–19:00	9:00–9:00 Day 2	All Moments	9:00–11:00	9:00–19:00	9:00–9:00 Day 2	All Moments
β_0_																
Anterior thigh	0.44	0.46	0.33	0.47	0.5	0.6	0.6	0.4	15	19	17	18	25	28	31	26
Knee	0.59	0.64	0.71	0.63	0.8	0.8	0.8	0.5	19	20	17	20	38	38	39	34
Anterior leg	0.49	0.64	0.73	0.54	0.4	0.5	0.5	0.3	12	12	11	15	21	25	26	23
Posterior thigh	0.63	0.53	0.69	0.59	0.5	0.5	0.6	0.4	9	12	10	12	16	18	21	18
Posterior knee	0.58	0.40	0.42	0.43	0.6	0.5	0.6	0.4	15	16	15	17	25	24	25	24
Posterior leg	0.73	0.78	0.69	0.68	0.5	0.5	0.5	0.4	7	8	9	10	18	18	19	18
β_1_																
Anterior thigh	0.41	0.31	0.36	0.43	0.08	0.08	0.09	0.06	18	25	22	23	32	33	36	32
Knee	0.52	0.60	0.77	0.60	0.12	0.11	0.12	0.07	27	24	17	24	46	44	47	41
Anterior leg	0.42	0.61	0.68	0.55	0.06	0.07	0.07	0.05	18	16	16	18	28	31	31	28
Posterior thigh	0.53	0.38	0.64	0.58	0.08	0.08	0.10	0.06	14	15	16	15	25	24	31	25
Posterior knee	0.57	0.28	0.39	0.33	0.09	0.08	0.08	0.06	19	21	19	23	31	30	29	30
Posterior leg	0.59	0.74	0.69	0.69	0.07	0.08	0.08	0.05	12	9	11	12	23	22	23	22

## Data Availability

The datasets generated and analyzed during the current study are available from the corresponding authors on reasonable request.
